# At What Latency Does the Phase of Brain Oscillations Influence Perception?

**DOI:** 10.1523/ENEURO.0078-17.2017

**Published:** 2017-06-07

**Authors:** Sasskia Brüers, Rufin VanRullen

**Affiliations:** 1 Université de Toulouse Paul Sabatier, 31062 Toulouse cedex 9, France; 2 Centre de Recherche Cerveau et Cognition, CNRS, UMR 5549, BP 25202, 31052 Toulouse Cedex Toulouse, France

**Keywords:** EEG, oscillation, phase modulation

## Abstract

Recent evidence has shown a rhythmic modulation of perception: prestimulus ongoing electroencephalography (EEG) phase in the θ (4–8 Hz) and α (8–13 Hz) bands has been directly linked with fluctuations in target detection. In fact, the ongoing EEG phase directly reflects cortical excitability: it acts as a gating mechanism for information flow at the neuronal level. Consequently, the key phase modulating perception should be the one present in the brain when the stimulus is actually being processed. Most previous studies, however, reported phase modulation peaking 100 ms or more before target onset. To explain this discrepancy, we first use simulations showing that contamination of spontaneous oscillatory signals by target-evoked ERP and signal filtering (e.g., wavelet) can result in an apparent shift of the peak phase modulation towards earlier latencies, potentially reaching the prestimulus period. We then present a paradigm based on linear systems analysis which can uncover the true latency at which ongoing EEG phase influences perception. After measuring the impulse response function, we use it to reconstruct (rather than record) the brain activity of human observers during white noise sequences. We can then present targets in those sequences, and reliably estimate EEG phase around these targets without any influence of the target-evoked response. We find that in these reconstructed signals, the important phase for perception is that of fronto-occipital ∼6 Hz background oscillations at about 75 ms after target onset. These results confirm the causal influence of phase on perception at the time the stimulus is effectively processed in the brain.

## Significance Statement

When investigating the relationship between ongoing electroencephalography (EEG) oscillations and perception in humans, most studies report a peak influence of the EEG phase before stimulus onset. However, we should also be able to measure these effects poststimulus, when the target is actually processed by the brain. First, we use simulations to show that a combined influence of the target-evoked potential and filtering can explain the lack of poststimulus phase modulation in typical studies. Crucially, we then introduce a paradigm to uncover the true latency at which phase influences perception. The white noise paradigm allows us to model background oscillations without target-evoked potentials. For the first time, we show that a θ-band ongoing oscillation influences perception ∼75 ms after target onset.

## Introduction

Instead of continuously processing the incoming visual information, the “perceptual cycles” hypothesis suggests that our brain relies on a rhythmic sampling of the input. Ongoing oscillations have been proposed as a mechanism through which our environment is sampled from 5-15 times a second (VanRullen, 2016b; VanRullen and Koch, 2003). At the neuronal level, this is realized through a biasing of the neuronal firing by local field potential (LFP) phase in various frequency bands (Fries et al., 2002; Jacobs et al., 2007). This results in periodic fluctuations of the excitability of the cortex (Fries, 2005; Lakatos et al., 2008): spikes in sensory cortices are more likely to occur at a given phase of LFP α oscillations than at the opposite phase (Haegens et al., 2011; Haegens et al., 2015).

At the global level, this relationship between excitability and phase has been studied using transcranial magnetic stimulation (TMS) and electroencephalography (EEG): an illusory percept (a “phosphene”) was most likely to occur when the TMS pulse was applied at a certain phase of the ongoing EEG oscillation than at the opposite phase (Dugué et al., 2011). Moreover, ongoing EEG oscillations have also been linked with visual perception: for example, the ∼7 Hz ongoing oscillation phase over fronto-central channels could account for 16% of the variability in the detection of near-perceptual threshold peripheral targets (Busch et al., 2009). Various studies have related ongoing EEG phase to behavioral and perceptual outcome using near-perceptual threshold target detection with attentional manipulation (Busch and VanRullen, 2010) or without it (Nunn and Osselton, 1974), using contour integration tasks at perceptual threshold (Hanslmayr et al., 2013), using suprathreshold stimuli detection (Callaway and Yeager, 1960; Mathewson et al., 2009), using eye-movement initiation (Drewes and VanRullen, 2011) and mislocalization (McLelland et al., 2016) or using a temporal illusion (Chakravarthi and VanRullen, 2012). Crucially, most of these experiments find the effects of the ongoing θ and/or α-phase during the prestimulus period, sometimes peaking even up to 200 ms before stimulus onset (for a review, see VanRullen, 2016b).

Superficially, this prestimulus effect might seem counter intuitive: the critical phase for perception should be the one present in the cortex during stimulus processing. If oscillatory signals are consistent over time, this phase influence may of course be visible several hundred of milliseconds before, but why does it vanish as stimulus onset approaches? The event-related potential (ERP) evoked after target presentation could be causing this seemingly contradictory finding: this relatively high-amplitude signal with similar phase values on every trial is likely to obscure any difference in background oscillatory phase of perceived and unperceived trials that would be present after stimulus onset, and thus apparently “push back” in time our ability to detect any such phase difference. This is especially true when considering window-based time-frequency analysis methods (a necessary step in oscillatory phase analysis), which will smear the effect in time (Lakatos et al., 2005; VanRullen, 2011, 2016a; Hanslmayr et al., 2013). Can we overcome these biases, and uncover the exact latency at which the phase of ongoing EEG oscillations modulates perception?

First, we use simulations to control the exact timing and oscillatory frequency at which a phase modulation of perceptual outcome is inserted into an artificial EEG dataset. We then assess the latency at which a significant phase difference between two conditions can be detected, and verify that this latency can be vastly underestimated.

Secondly, we introduce the white noise (WN) paradigm, based on linear-systems analysis (Marmarelis and Marmarelis, 1978) and reverse correlation methods (Ringach and Shapley, 2004), which are used to characterize the systematic relationship between visual stimulation and brain response, i.e., the impulse response function (IRF). This approach has been applied to EEG to measure evoked potentials (VESPA; Lalor et al., 2006) or to reveal “perceptual echoes” (VanRullen and Macdonald, 2012). Once extracted, a simple convolution with these IRFs can be used to model the brain’s EEG response to any new WN sequence presented (Ringach and Shapley, 2004). Within these new WN sequences, we also embedded near-perceptual threshold targets, which had a medium gray luminance level with the same properties as any other frame in the sequence. Accordingly, they did not affect the convolution result. Thus, we effectively removed the target-evoked response from our signal, and only modeled the background oscillations. Therefore, we could measure the real latency at which this background oscillatory phase impacts visual perception.

## Materials and Methods

### Measuring phase differences

Both the simulation and the experimental paradigm relied on quantifying the phase difference between two conditions, whether simulated or based on the actual behavioral outcome of human observers. To this end, we used the phase opposition sum (POS) measure (VanRullen, 2016a), which relies on the intertrial phase clustering (ITPC, also phase locking value or factor; Tallon-Baudry et al., 1996; Lachaux et al., 1999) and is computed as follows:

$$ITPC_{\left(t,f\right)}=\left|\frac{1}{n}\overset{n}{\underset{k=1}{\sum }}e^{\left(i\varphi_{k}(t,f)\right)}\right|$$

The ITPC is, for a given time point *t*, and frequency of interest f, the norm of the complex average across n trials of the vector with unit length and phase φ. Consequently, the POS (VanRullen, 2016a) was computed as the sum of the ITPC for each condition corrected by subtracting the ITPC for the combined conditions, as follows:

$$POS=ITPC_{A}+ITPC_{B}-2ITPC_{all}$$

Theoretically, the POS takes values between 0 and 2. A value of 0 arises when phase distributions for each condition are fully random (uniform) or when both conditions have their phases locked to the same angle. A value of 2 represents a perfect phase opposition between the two conditions, that is, all trials with behavioral outcome A have the same phase angle, and all trials with outcome B have the opposite angle (with no ITPC across the entire set of trials). Intermediate POS values reflect a partial phase opposition (a more plausible scenario) in which the phase modulates the probability of outcome A versus B. These theoretical values rely on the assumption that there is a perfect uniform sampling in the phases across all trials. Since this is unlikely to be the case in real experiments, it is better to compare the POS value to a corresponding distribution of surrogates POS values obtained by shuffling the labels between conditions. This will account for any bias in the underlying ITPC across all trials.

The POS was computed separately for each dimension of the dataset (time, frequency, channels, and subjects). The strength of the effect was evaluated at each of these dimensions by creating a surrogate distribution of 1000 permuted POS values through shuffling of the labels between conditions (i.e., creating random partitions). The mean and variance of the surrogate distribution were extracted to compute the *z* score of the observed POS, which was then transformed into a *p* value using the normal cumulative distribution function (for a description of this method and a comparison with other measures, see VanRullen, 2016a).

## Simulations

In a first part, we used simulations of artificial datasets to look at how the ERP shape and frequency content, coupled with the time-frequency decomposition, influenced the latency at which a phase difference between two conditions could be detected, depending on the frequency of the phase modulation.

### Creating artificial datasets

**Figure 1.**
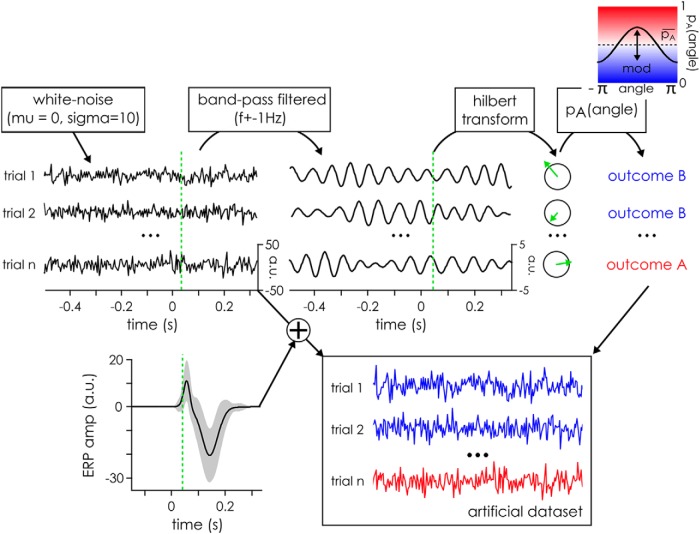
Illustration of artificial datasets creation for the simulation. The artificial signal was initialized using WN drawn from a Gaussian distribution with μ = 0 and σ = 10 arbitrary units. These random data were then bandpass filtered at the frequency of interest plus or minus 1 Hz, and a Hilbert transform was applied to extract the phase at 40 ms after time 0, the time of target presentation. The phase angle at this time was then used to separate the trials between outcome A and B, with a given probability following a cosine function. Finally, an outcome independent ERP wave form (with slight random variations between trials) was added to each trial’s signal to create the final artificial dataset.

To evaluate the full extent of the effect, we systematically varied the frequency at which the phase modulation was inserted from 3.99 to 100 Hz in 24 logarithmically spaced steps. For each of the 24 frequency of interest, 100 artificial datasets (corresponding to the “subjects” in traditional EEG experiments) were created using an approach similar to that described in VanRullen (2016a). First, the background electrophysiological signal was simulated by creating 500 WN sequences drawn from a Gaussian distribution with a μ of 0 and a σ of 10 arbitrary units (Fig. 1). These sequences lasted 3 s ([−1.5 to 1.5 s]) and had a sampling rate of 500 Hz.

Once the artificial datasets had been generated, a phase modulation between two experimental conditions (i.e., trial groups) was artificially created using the phase of the frequency of interest at an arbitrarily chosen time point (40 ms after target onset; Fig. 1, green line). This phase was extracted by filtering the datasets at the frequency of interest and then applying a Hilbert transform. It was then used to assign an experimental condition label to each trial. Each of the two conditions was equally likely to occur overall (i.e., mean probability $\bar{p_{A}}$ of outcome A was equal to the probability $\bar{p_{B}}$ of outcome B). However, the likelihood of a trial outcome was modulated using a cosine function of the phase angle at the critical time, with a modulation depth (denoted as *mod* in the following equation) fixed at 0.4 (arbitrarily defined parameters). It was computed as follows:

$$p_{A(angle)}=\overset{¯}{p_{A}}+mod\;\cdot \;cos(angle)$$

In our case, this means that for trials at phase 0, there was a 70% chance of the trial yielding outcome A, while at the phase π, the trial had 70% chance to yield outcome B. Finally, an ERP was added to each trial. Both conditions had an ERP drawn from the same process, which was composed of a P1 and an N1 waves with (arbitrarily defined) parameters. The exact shape of the ERP differed slightly on each trial, as the parameters were drawn from normal distributions with known mean and σ defined for our purposes as follows (Fig. 1, average representation): the P1 mean amplitude was fixed at 20 units (amplitude σ of 5) and its mean peak latency at 65 ms (peak latency σ of 10 ms) with a mean duration of 50 ms (duration σ of 10 ms). The N1 mean amplitude was fixed at 30 units (amplitude σ of 10) with a mean peak latency of 155 ms (peak latency σ of 25 ms) and a mean duration of 130 ms (duration σ of 25 ms). The scale of the arbitrary units was defined with respect to the standard deviation of the originally generated WN signal, equal to 10 units. The final signals with the added ERPs were then used as the artificial dataset to analyze.

In the main simulations, Gaussian WN, with equal power at all frequencies, was used as a proxy for brain activity, as described above. Using pink noise instead of WN could be deemed more biologically plausible; however, pink noise (or 1/f) signal is characterized by a power spectrum decreasing as a function of frequency, meaning that differences of signal-to-noise ratio between frequencies could then have confounded our ability to detect phase effects. Nonetheless, we also performed control simulations using pink noise, to verify that the conclusions held with more biologically plausible input data. The same parameters as above were used. These control simulations gave comparable results, which are not presented here for the sake of brevity.

### Extracting the latency of the phase difference

Once the final artificial datasets had been created, the time-frequency information was extracted using a wavelet decomposition, varying the numbers of cycles (logarithmically) from three cycles at 3Hz to 8 cycles at 100 Hz for each of the 50 (logarithmically spaced) frequency steps. The time course of the phase modulation was evaluated by looking at the time course of significant phase opposition using the *p* values extracted (see above, Measuring phase differences). For the purpose of these simulations, we assume that the rhythmic modulation frequency is known, and we aim to derive the latency of the effect. To this end, we restricted our analysis in time and frequency to an analysis window spanning 800 ms around the true latency of the phase modulation (i.e., from −360 to 440 ms) at the actual frequency at which the phase modulation had been introduced in the dataset. For each of the 100 artificial datasets, the time course of significance of the POS was evaluated by only keeping *p* values reaching or exceeding a Bonferroni threshold computed so as to correct for multiple comparisons across the 170 time points of the analysis window. This was taken as evidence for a significant phase difference between the two conditions at that particular latency. The time courses for each of the artificial datasets were then aggregated by computing the percentage of the simulated datasets which showed a significant POS at each time point. From this, we also computed the mean latency of the largest cluster for each of the time courses of the 100 datasets as a measure of central tendency. These latencies were then aggregated across all artificial datasets by looking at the confidence intervals (CIs) for the median latency of the phase modulation across artificial datasets, using the following formula by McGill et al. (1978), where q1, q2, and q3 are, respectively, the 1st, 2nd (or median), and 3rd quartile of the distribution and *N* is the number of artificial datasets.

$$95\%CI=q2\pm 1.7*\frac{1.25(q3-q1)}{1.35\sqrt{N}}$$

We also computed the significance of the temporal distortion effect using a Wilcoxon sign rank test comparing the observed median latency to the true modulation latency of 40 ms, using an α level of 0.01.

## The WN paradigm

In the experimental part, we introduce a paradigm based on linear-systems analysis methods, to help us uncover the true latency at which phase influences perception, avoiding the pitfalls induced by target-evoked responses.

### Participants, stimuli, and procedure

Twenty-one subjects (mean age of 28.04 years, SD: 3.97 years, 23–39 years old) took part in the experiment after giving written informed consent. One subject was removed due to technical issues during the EEG recording, thus 20 subjects were analyzed (10 women, 15 right handed, normal or corrected-to-normal vision, no history of epilepsy). The experiment consisted of two testing sessions of ∼1 h each, composed of eight blocks of 48 WN (random luminance) sequences (WN). Each WN sequence lasted 6.25 s and had, on average, a flat power spectrum between 0 and 80 Hz. They were presented in a peripheral disk at 7° of visual angle from fixation point and subtending 7° of visual angle on a cathode ray monitor (resolution of 640 by 480 pixels and refresh rate of 160 Hz) situated 57 cm from the chin rest. In both sessions, participants had to detect near-perceptual threshold targets embedded in the stimuli sequences. In each trial, there were two to four targets composed of a lighter disk at the center with a darker surrounding annulus. They were presented within the peripheral flashing disk on a medium gray background disk for one frame only. The mean luminance of the target was always a medium gray, identical to the mean gray level of the WN sequence; only the contrast within the target was manipulated. A staircase procedure was conducted over the first 100 targets (i.e., ∼30 trials) of each session using the *Quest* function (Watson and Pelli, 1983) in the PsychToolBox (Brainard, 1997). The contrast between the darker annulus and the lighter inner circle was adjusted to converge to the luminance contrast at which people perceived 50% of targets on average. This contrast was then kept for the remainder of the session. Session 1 data revealed that the visibility of the target was influenced by immediately surrounding luminance values (see below, Classification image). This influence could have masked the (potential) effect of oscillatory phase. Therefore, in session 2, we decided to remove any luminance fluctuations around targets, setting 14 frames before (i.e., 87.5 ms) and 11 frames after (i.e., 68.75 ms) the target to the same medium gray value.

Using a similar design and protocol, we ran a control experiment to assess whether the fluctuation-free periods could be used by subjects (*N* = 6) to detect the targets. The only difference was that for 1/3^rd^ of the suppressed luminance time windows no targets was presented, creating “catch trials” of sorts. As with the main experiment, any button press after a target (from 150 to 800 ms) was counted as a hit. The same response window was used for catch trials: any button press within this 650-ms time window after the moment where a target would have been was counted as a false detection. The percentage of catch trials where a subject pressed the button was computed, similarly to the percentage of detected targets. Separately, we also assessed the false alarm rates of subjects, i.e., button presses falling outside of response time windows for either presented targets or catch trials. To get an estimate of the false alarm rate that is comparable with the other detection rates, we counted the number of 650 ms long time windows outside of target times and divided the number of false alarms by this number.

### EEG recording and preprocessing

During the first session, the EEG to WN sequences was recorded using a 64-channels Biosemi system (1024 Hz sampling rate) with three external ocular channels recording the electrooculogram. Only the behavioral responses (to new WN sequences) were recorded in the second session. The preprocessing of the EEG data from session 1 was conducted using the EEGlab toolbox (Delorme and Makeig, 2004) in Matlab, with the following steps: (1) rejection and interpolation of noisy channels if necessary, (2) down-sampling of the EEG signal to 160 Hz to match the presentation rate of stimuli and thus facilitate the cross-correlation of the two signals, (3) notch filtering (between 47 and 53 Hz) to remove any artifacts due to power line, (4) average-referencing, (5) high-pass filtering (1 Hz) to remove any drifts in the signal, (6) creating epochs from −0.25 to 6.5 s around each WN sequence, (7) removing the baseline, i.e., the mean activity from −0.25 to 0 s before trial onset, and (8) manual artifact rejection where whole epochs were removed (as needed) to get rid of eye blinks and muscular artifacts. Once the data had been preprocessed, the IRF (also called VESPA by Lalor et al., 2006; or perceptual echoes by VanRullen and Macdonald, 2012) were extracted by cross-correlating the preprocessed EEG data with the WN luminance sequences, yielding 64 IRFs for each of the 20 subjects (one IRF for each EEG channel). These functions were then used to reconstruct the brain activity (“reconstructed EEG”) to the new WN sequences presented in session 2, by convolution of the IRF with the WN. The target luminance (medium gray) was included in the WN sequence used to reconstruct the EEG. However, this value was no different from the surrounding values in the sequences, and thus there was no ERP evoked by the target in the reconstructed EEG. In session 2, the same random sequences (different from those in Session 1) were presented to all subjects in a randomized order so as to compare the visibility of the same targets across all subjects. This reconstructed EEG was epoched around each of the 821 presented targets ([−800 to +794 ms]). Finally, a wavelet transform was applied to extract the oscillatory characteristics of the reconstructed EEG at each frequency band (two to eight cycles, 50 log-spaced frequencies from 3 to 100 Hz). Note that we limited our analysis to frequencies below 80 Hz as our signal was sampled at 160 Hz (Nyquist frequency limit).

### Measuring the phase difference between conditions

Further analyses of the phase differences between detected and undetected targets were evaluated only on the reconstructed EEG signals using the phase opposition method (see above, Measuring phase differences). Before extracting the z-scores, we computed the grand average POS values (whether real or permuted) by first summing the POS across electrodes and subjects to aggregate information along these dimensions. To increase the robustness, this step was repeated for the surrogate POS by randomly selecting (without replacement) surrogates for each subjects and summing across subjects again, a large number of times, thus yielding 100,000 grand average POS surrogates values. Using these grand averaged POS values, the z-scores and *p* values were then extracted as previously described (see above, Measuring phase differences). A false discovery rate (FDR) correction was applied across frequency and time points, using an α level of 0.05. Once a time-frequency region of interest was found, we extracted the topography of the effect by going back to single channel data and summing the phase opposition values across subjects and the time and frequency points composing the largest significant cluster. Here, again, to increase the robustness, we randomly selected (without replacement) surrogates for each subject and summed the POS across subjects again. This was conducted 10,000 times before extracting the z-scores as previously described.

### Measuring phase-dependent performance

To evaluate the strength of the effect and compare it with previous reports (Busch et al., 2009), the performance variability attributable to phase changes was computed on the fronto-central channel. The normalized hits ratio was extracted, for each subject and for each of the 11 phase bins as the proportion of hits in each bin, normalized by the mean performance of the subject. These were then averaged across subjects. A cosine fit was applied to the data and the resulting amplitude is reported as the amount of performance modulation for each study.

### Classification image

We sought to test whether the luminance values of the WN sequences at any specific time point around the target time had an impact on perception. To this end, we used the classification image method (Ahumada, 2002), which can help identify which stimuli parameters have an impact on performance. For both sessions, we computed the mean luminance values across trials for hits and missed targets for each subject separately. An independent samples *t* test was then applied for each subject to compare the distribution of luminance values for detected versus missed targets. A FDR correction was applied across time and subjects to correct for multiple comparisons in each of the sessions.

### Evoked response

We also wanted to confirm that the reconstructed EEG did not in fact show any sign of an evoked response to the target presented in the WN sequence. We computed the ERP for the EEG recorded in session 1 (as a control) and for the reconstructed EEG for session 1 and session 2. This was done by first removing the baseline activity ([−200 to 0 ms]) to each time course and then averaging the different signals across trials and subjects. Because the signal was visual in nature, we looked at the ERP over the central parieto-occipital channel (POz).

### Measuring the correlation between recorded EEG and reconstructed EEG

To test how well the reconstructed EEG modeled the recorded EEG, we used the data from session 1, for which the recorded EEG was available. First, the total number of trials for each subject (which was variable due to artifact rejection) was sub-sampled to the trial count available for the subject with the fewest trials. Then, using a 10-fold cross-validation strategy, the model (i.e., IRF) was computed on 90% of trials, and consequently used to reconstruct the EEG for the remaining 10% of trials. This reconstructed EEG was then correlated (using the Pearson correlation) with the EEG actually recorded on the remaining 10% of (independent) trials. This ensured that both the recorded and reconstructed EEG were available for the same sets of trials, while avoiding any circularity in the analysis: using the same trials to compute the IRF and the reconstructed EEG could have led to spuriously high correlations between reconstructed and recorded EEG. Moreover, this also reduced the influence of a sampling bias: using a cross-validation strategy in correlating the signals allowed us to get a better estimate of the true underlying correlation between the two signals.

We also tested whether the EEG modeled using the IRF was more accurate for certain frequency bands by correlating the two signals (with the same cross-validation approach) filtered in five different frequency bands (Finite Impulse Response filter, δ: 2–4 Hz; θ: 4–8 Hz; α: 7–14 Hz; β: 14–28 Hz; γ: 30–60 Hz).

Since the correlation coefficients were not normally distributed (as revealed by a one-sample Kolmogorov-Smirnov test), a Fisher Z transform was applied to the data. It was computed as follows:

$$Z=0.5*ln\left(\frac{1+r}{1-r}\right)$$

Consequently, the mean transformed coefficients across all repetitions were extracted for each subject and channel and a one sample *t* test against zero was applied. The *p* values were corrected for multiple comparisons using a FDR correction across channels. To extract the correlation coefficient values for plotting purposes, the inverse Fisher Z transform was applied. Only the correlation coefficients at the strongest channel are presented across subjects. Note that using a nonparametric test (instead of a Fisher Z transform and a *t* test) gave equivalent results.

As a comparison, we also tested how much of the variability in the signal could be explained by the ERP to the target embedded in WN. Here, we used target ERPs (rather than IRF) as a model for the target-evoked activity. Finally, for comparison purposes, we also wanted a measure of how “noisy” EEG data normally is (i.e., in typical ERP paradigms without ongoing WN sequences). We tested this using a separate dataset from Busch et al. (2009), in which isolated targets were presented on a static background. The target-evoked ERP was again used as a model of evoked EEG activity. In both conditions (ERPs from targets embedded in WN; ERPs from isolated targets), the ERPs were extracted on 90% of the trials and then convolved with a sequence of target onsets for the remaining 10% of trials, in a 10-fold cross-validation approach. Consequently, the same correlation method between recorded and reconstructed EEG (modeled here by the ERP) was applied as described above for the IRF-based EEG reconstruction model.

## Results

### Simulations

Using artificially created datasets with a true phase modulation introduced at 40 ms after stimulus onset, we evaluated how early a significant phase opposition would be detected. We measured the time course of the *p* values of the POS (see Materials and Methods), aggregated across 100 simulated datasets (for more reliability). In particular, we tested various frequencies of phase modulation from 4 to 100 Hz to see if this factor influenced the latency at which a significant effect could be measured.

**Figure 2.**
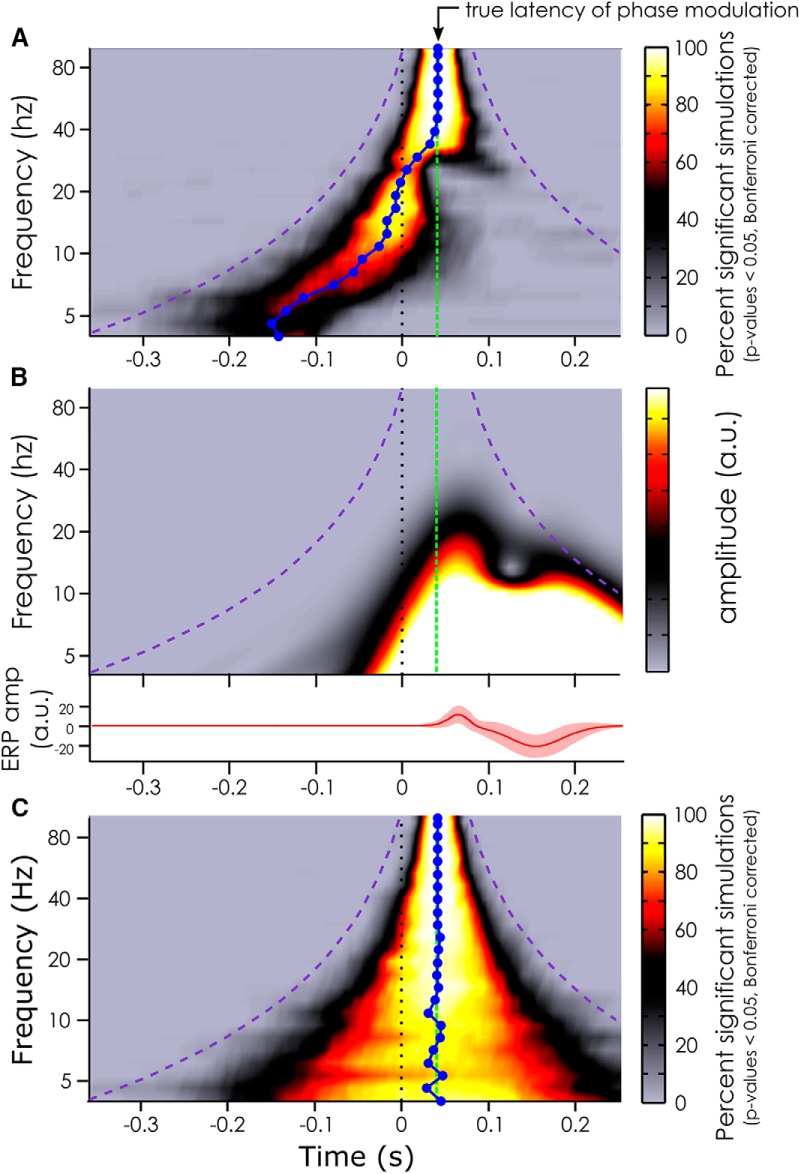
Simulation results. ***A***, Median latency (blue line) at which phase modulation effects can be measured depending on the frequency of the phase modulation introduced at 40 ms (true latency represented by the green dashed line) when the ERP is included in the artificial datasets. The color bar indexes the percentage of significant datasets at each time point after Bonferroni correction. The purple dashed line represents the outer edge of the window function (Morlet wavelets). ***B***, Representation of the evoked response (lower panel) included in the artificial data in ***A***, and its time-frequency content (upper panel). The color bar represents the oscillatory amplitude (arbitrary unit) at each frequency and time point. **C**. Same analysis as in ***A*** but without ERP included in the artificial datasets. Note the absence of temporal latency distortion in this case. Note also that the temporal smearing created by the window function is slightly shorter than the window duration (i.e., it does not reach the purple line) as Morlet wavelets have Gaussian tapers on either end.

At all frequencies of phase modulation, we did find evidence for a phase difference between conditions (as expected given that this phase modulation had been explicitly introduced in each dataset). But at frequencies of the phase modulation below 30 Hz, the observed phase modulation appeared to peak well before the true latency of the phase modulation. At lower frequencies (roughly below 20 Hz; Fig. 2*A*), this effect was even visible in the prestimulus time window. This temporal displacement seemed to be frequency dependent: the lower the frequency, the earlier the phase modulation was detected (Fig. 2*A*). In fact, the median latency of observed phase opposition effects was significantly different (Wilcoxon sign rank test) from the true phase modulation (at 40 ms) at all frequencies between 3.99 and 39.44 Hz. At 3.99 Hz, the median measured phase modulation latency peaked at −143 ms in the prestimulus window (95% CI: [−151 to −135 ms]). At 39.44 Hz, the median latency of the effect was at +37.5 ms, i.e., 3 ms before the true effect (95% CI: [35–39 ms]). At frequencies higher than 40 Hz, the median latency of the observed phase modulations effects was not significantly different from the true latency.

Interestingly, these temporal displacements seemed to be closely linked to the time-frequency content of the ERP: the latencies at which the phase difference could be measured were pushed back in time by an amount commensurate with the temporal spread of ERP spectral power at the corresponding frequency (Fig. 2*B*). As a matter of fact, when the ERP was removed from the simulated data, and all the analyses were performed again, the median latency of the phase modulation was not shifted (Fig. 2*C*). The only effect seen in this case was a smearing around the true latency of the phase modulation due to the window-based time-frequency decomposition. In fact, this smearing of information can account for the specific shape of the temporal displacement created by the ERP: there is a strong correlation (Pearson correlation, *r* = 0.81, *p* < 0.05, 95% ci for *r* = 0.799–0.827) between the error in the measured latency of the phase modulation effects (Fig. 2*A*) and the length of the window function at that frequency. Here, the smearing had a chimney-like shape typical of wavelet analysis: the smear of information varied from 750 ms at lower frequencies (at 4 Hz: window function of three cycles of 250 ms each) to 80 ms at the highest frequency (100 Hz) around the region of interest. The absence of latency distortion in the absence of ERP confirms the notion that it is the stimulus-evoked activity (and its temporal smear caused by any window-based time-frequency decomposition) that is likely responsible for the prevalence of EEG phase modulations reported to peak in the prestimulus time window in the relevant literature (for a review, see VanRullen, 2016b).

We showed that a phase difference between the two conditions in our artificially created datasets could be measured well before the latency at which the actual phase modulation was introduced. This temporal displacement could be quite dramatic at lower frequencies, with peak effects being apparently pushed by almost 200 ms. It was evident that the time-frequency content of the ERP was responsible for this apparent shift for at least two reasons: first, the measured latency of the phase modulation directly followed the left edge of the time-frequency content of the ERP (Fig. 2*B*); second, the temporal displacement disappeared when the analysis was replicated without an ERP (Fig. 2*C*).

Our simulations highlight a large uncertainty regarding the latency of EEG phase modulation. Importantly, this uncertainty is expected whenever ERPs are present and signal filtering is used, and would appear to be unavoidable for any experimental measure of phase-dependent perception. Is there a way, then, of uncovering the true latency at which ongoing phase affects stimulus detection?

### The WN paradigm

**Figure 3.**
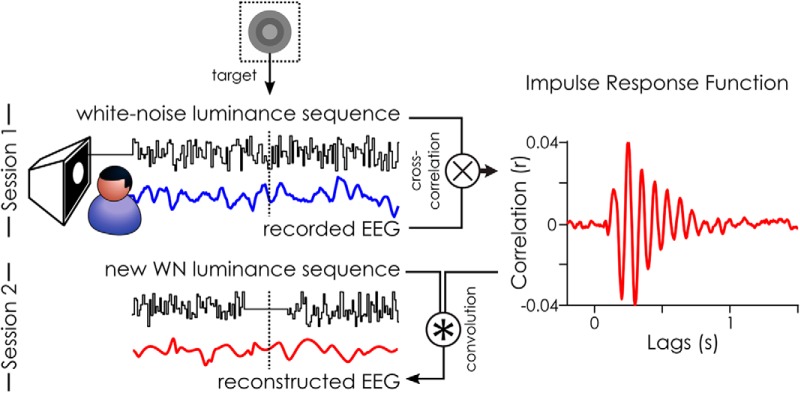
White-noise (WN) paradigm. The IRF to WN sequences can be extracted by cross-correlating the stimuli sequence with the recorded EEG: here, an example IRF is shown from one subject on electrode POz. This is what we did in the first session of our experiment. This IRF can, in turn, be used to reconstruct the brain activity (reconstructed EEG) to any new WN sequence by convolution. This was done in the second session of the experiment. The stimulus fluctuations around the target were removed in the second session to avoid any target masking by the luminance (Fig. 5).

Here, we sought to experimentally investigate the time course of phase effects using the WN paradigm, i.e., using WN sequences to constrain ongoing brain oscillations in a predictable manner. The IRF can serve to model the relationship between fluctuations of luminance values in the sequence and the brain response at different delays (Fig. 3; Ringach and Shapley, 2004; Lalor et al., 2006; VanRullen and Macdonald, 2012). It is extracted by cross-correlating WN sequences with the concurrently recorded EEG. These IRFs can then be used to reconstruct the brain activity to any new WN sequences presented. This reconstruction was done by convolving the IRFs with the new WN sequences presented in the second session (Fig. 3). We call this signal the reconstructed EEG: a model of the brain activity in response to the WN sequences.

Before investigating the time course of phase effects, we verified that our new paradigm met specific requirements. First, we sought to quantify the correlation between the modeled brain activity (the reconstructed EEG) and the real data (recorded EEG).

#### Correlating the reconstructed and recorded EEG

We first quantified the similarity between the recorded EEG (in session 1) and the EEG reconstructed using the IRF as a model of the background activity in the EEG. This was achieved by correlating the two signals using a 10-fold cross-validation approach: the IRF was computed on 90% of trials, and then used to model the EEG response for the remaining 10% of trials, which we then correlated with the recorded EEG.

A one-sample *t* test revealed that the distribution of the mean (Z transformed) correlation coefficients using the raw signal was significantly different from 0 on all channels, with the mean correlation coefficient across subjects reaching a maximum on electrode Oz with a r of 0.091 (*t*_(19)_ = 7.78, *p* = 2.51*10^−7^, 95% CI for *r* = 0.066–0.115). We also investigated whether filtered signals would yield a better correlation depending on the frequency band. We found that the correlation strength was strongest for the α-band on channel Oz (mean *r* = 0.163, *t*_(19)_ = 8.21, *p* = 1.14*10^−7^, 95% CI for r: 0.121–0.204), closely followed by the θ-band on channel POz (mean *r* = 0.125, *t*_(19)_ = 13.69, *p* = 2.69*10^−11^, 95% CI for r: 0.105–0.144). The other frequency bands had lower correlation coefficients, with, respectively, a mean *r* of 0.058 (channel POz) for the δ-band (*t*_(19)_ = 7.28, *p* = 6.60*10^−7^, 95% CI for r: 0.041–0.075), a mean *r* of 0.071 (on channel Oz) for the β-band (*t*_(19)_ = 7.80, *p* = 2.42*10^−7^, 95% CI for r: 0.052–0.090) and a mean *r* of 0.027 (on channel POz) for the γ-band (*t*_(19)_ = 6.16, *p* = 6.37*10^−6^, 95% CI for r: 0.018–0.037).

**Figure 4.**
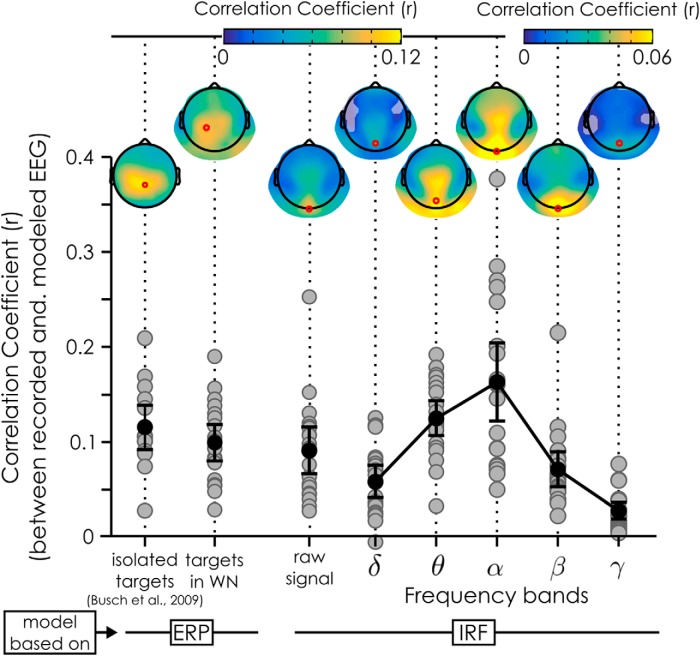
Correlation between reconstructed and recorded EEG. Two models of brain EEG activity were tested: one based on the ERP to targets (left) and the other based on the IRF to WN sequences (right). In both cases, we systematically correlated the prediction of the model with the single trial recorded EEG. For the ERP models, the **“**isolated targets**”** coefficients are based on the dataset presented in Busch et al. (2009), while the **“**targets in WN**”** are from our own dataset, with the ERP extracted relative to the targets embedded in WN (Fig. 5*C*). For the IRF models, we used the IRF to model the brain response to the WN sequences, and then correlated reconstructed and recorded EEG data using the raw signals, as well as signals filtered in different frequency bands (δ: 2–4 Hz; θ: 4–8 Hz; α: 7–14 Hz; β: 14–28 Hz; γ: 30–60 Hz). The gray dots represent the mean coefficient for each subject (1 dot per subject) across cross-validation runs at the maximum electrode (red dot on the topographies); the error bars represent the 95% CI of the mean (black dot) coefficient across subjects. The topographies represent the mean correlation coefficients across subjects and cross-validation runs. Shaded areas represent channels not significant after FDR correction. Note the difference of color scales for the β and γ correlation coefficients relative to the other topographies.

To better evaluate the quality of our EEG reconstructions, we decided to run two control analyses, as a basis for comparison. First, we wanted to know how much of the variability in the signal could be explained using the ERP instead of the IRF as a model of the EEG activity to targets presented in WN (for details, see Materials and Methods, Measuring the correlation between recorded EEG and reconstructed EEG). Using the ERP as a model for target evoked activity led to a correlation strength on par with that obtained using the IRF as a model of background activity. The correlation coefficient reached its maximum across subjects over the left central parietal channel (CP1) with an r of 0.099 (*t*_(19)_ = 10.92, *p* = 1.25*10^−9^, 95% CI. for *r* = 0.080–0.118, see “targets in WN”; Fig. 4). Secondly, we also evaluated the amount of signal variability that can be explained by the ERP to isolated targets in a more “typical” visual-evoked potential paradigm (i.e., without the concurrent WN stimulation), using the data from Busch et al. (2009). This was done as a way to estimate the “noise” in a typical EEG setting, for comparison purposes. A one-sample *t* test revealed that the distribution of the mean (Z transformed) correlation across subject reached a maximum over channel (Pz) with an r of 0.115 (*t*_(13)_ = 9.57, *p* = 2.97*10^−7^, 95% CI for r: 0.089–0.141; Fig. 4, isolated targets). In conclusion, using the IRF as a model for reconstructing the EEG is a useful, although far from perfect (i.e., *r* < 0.2), characterization of the recorded EEG; although the obtained EEG reconstruction was clearly noisy, the reconstruction error was not much higher than that observed with ERP-based models of target-evoked activity.

#### Behavioral results

In both sessions, the participants had to detect targets embedded in the WN luminance sequences. The contrast of the target with regard to its medium gray background was adjusted to achieve 50% performance at the beginning of the experiment. The mean hit rate across the 20 participants for session 1 was 50.38% (SD: 9.17%) and 45.76% (SD: 10.88%) for session 2. For both sessions, the false alarm rate was relatively low with a mean false alarm rate of 1.65% (SD: 1.35%) and 1.65% (SD: 1.49%) for sessions 1 and 2, respectively. A control experiment revealed that the suppressed luminance fluctuations could not be used by subjects to detect the targets. The mean response rate to catch trials across subjects (i.e., suppressed luminance without targets presented) of 8.94% was not significantly different from the mean false alarm rate of 6.30% (Student’s *t* test, *p* = 0.646). Moreover, both were much lower than the mean detection rate across subjects of 52.80% (Student’s *t* test, *p* < 0.002).

**Figure 5.**
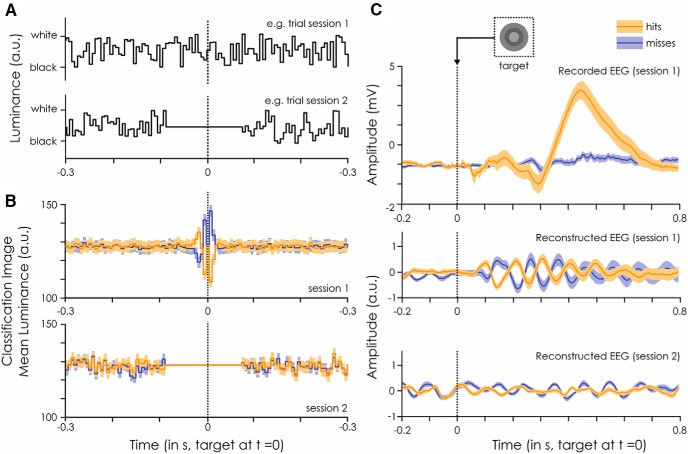
Classification image and evoked response. ***A***, Example stimuli sequences for session 1, when the EEG was recorded, and session 2, when only the behavioral response was recorded. The sequences are centered on the embedded target presentation (at *t* = 0 ms). ***B***, Classification image of the luminance values around the target (at *t* = 0 ms) for detected (orange) and missed (blue) targets. Darker colors represent the mean across subjects and trials. The lighter shades represent the standard error of the mean across subjects. The dotted line represents target presentation. Note that the same sequences were shown to all subjects in session 2. ***C***, ERP evoked by the detected (orange) and missed (blue) targets embedded within the WN sequences. These are computed separately for the recorded EEG for session 1 (top) and the reconstructed EEG for session 1 (middle) and 2 (bottom). Note the absence of visible target-evoked ERP in reconstructed signals for session 2 (bottom). While there seems to be a very strong phase opposition between hits and misses in the reconstructed EEG to session 1, this is likely to be an artifact of the strong relationship between luminance value and behavioral outcome illustrated in ***B***, rather than a direct relationship between phase and perception. Darker colors represent the mean across trials and the lighter shades represent the standard error of the mean across subjects. The dotted line represents target presentation.

**Figure 6.**
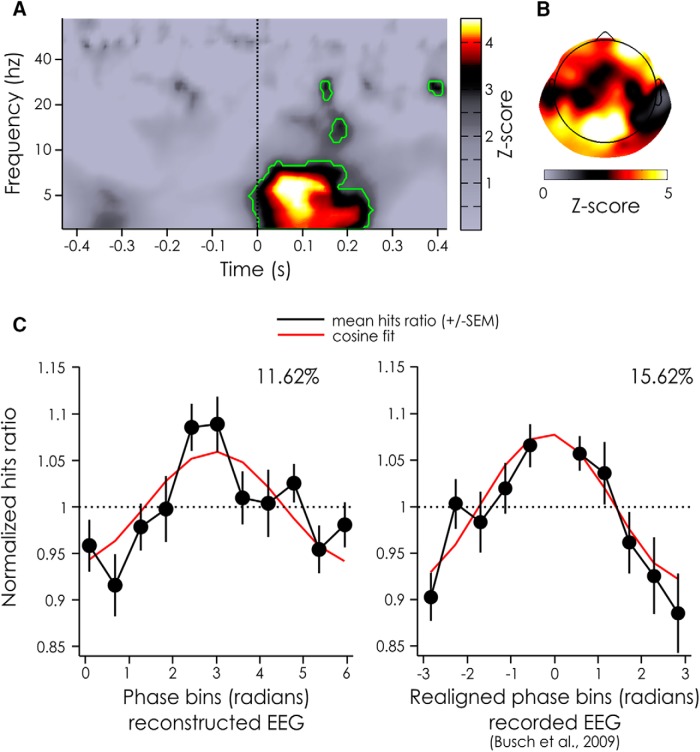
Z-score of POS for the reconstructed EEG and phase dependent performance. ***A***, Z-scores of the POS values aggregated across electrodes and subjects for the reconstructed EEG for each time and frequency point. Significant time points after a FDR correction (α = 0.05) are outlined in green. ***B***, Z-scores of the POS values aggregated across subjects for the reconstructed EEG at the frequencies and time points inside the largest significant cluster (from 3–8.17 Hz and from −12.5 ms to 250 ms, ***A***). ***C***, Normalized hits ratio depending on the phase bin for the reconstructed EEG at the peak significance (left hand side: 6.1 Hz, +75 ms). For comparison, previously published results linking detection performance and prestimulus phase of actually recorded (rather than reconstructed) EEG signals are replotted on the right hand side (7 Hz, −120 ms; taken from Busch et al., 2009). Both phase dependence curves are measured on the same fronto-central channel. Note that in our experiment the phase bins were not realigned between subjects, but that they were realigned (to produce peak performance at 0 for all subjects) for the experiment by Busch et al. (2009). The black line represents the mean across subjects (left hand side: 20 subjects, right hand side: 14 subjects), the bars represents the SEM, the red line represents a cosine fit, whose modulation amplitude (in %) is noted above the curve.

A classification image analysis of the data from session 1 showed that the luminance values immediately surrounding the target had a large impact on target detection. Systematically, higher luminance values just before and after the target led to decreased visibility while lower luminance values led to increased visibility (Fig. 5*B*). In session 1, all 20 subjects showed a significant difference in the mean luminance values between detected and missed target trials (independent sample *t* test for each subject, with FDR correction, all 20 peak *p* values below *p* < 0.0008), and this difference affected at least 3 separate time points for all subjects (Fig. 5*B*). For this reason, we decided to remove luminance fluctuations around the target. In the second session, 87.5 ms (i.e., 14 frames) before the target and 68.75 ms (i.e., 11 frames) after the target were replaced with medium gray values (Fig. 5*A*), the same medium gray used as the target’s background. In session 2, this manipulation effectively cancelled the apparent masking or facilitation effects observed in session 1 (Fig. 5*B*): 18 of the 20 subjects showed no significant luminance difference between hits and misses at any time point; the remaining two subjects showed a significant difference at only a single time point. Note that we also verified that all results described in the following section (Fig. 6) remained valid when these two subjects were discarded from the analysis.

The major advantage of using reconstructed EEG for our purposes is that there is no evoked response to the targets embedded in the stimulation sequences. This is because the luminance of the target frame (medium gray), which is taken into account when the convolution is done, is identical to the luminance of the surrounding frames. In other words, the presence of the target is invisible to the EEG reconstruction process (convolution). In fact, we can see that there is no evoked ERP to the target in the reconstructed EEG for session 2 (Fig. 5*C*).

#### Latency of measured phase modulation effects in the absence of ERP

Because the reconstructed EEG is virtually blind to the presence of target-evoked activity, we can estimate the background or “ongoing” signals around the time of target presentation. Thus, we can causally investigate the dynamics of the ongoing oscillations’ influence on perception, without having to restrict our analysis to the prestimulus window due to evoked response contamination.

We applied a standard procedure to evaluate the oscillatory “phase opposition” among groups of trials in which the target was detected versus missed (VanRullen, 2016a); but in this case, the input data were the reconstructed EEG (obtained by convolution with the IRF) rather than an actually recorded EEG signal. The phase of ongoing oscillations in the θ-band (∼6 Hz) seemed to be significantly different for detected versus missed targets, compatible with findings from previous studies (Busch et al., 2009). However, here the largest effect was present at ∼75 ms after target onset (*p* = 1.2242*10^−6^; Fig. 6*A*). A scalp topography revealed that phase opposition was maximal over frontal and occipital channels (Fig. 6*B*).

We then evaluated the amount of performance modulation that could be explained by the ongoing oscillatory phase at the peak of significance (i.e., 6 Hz and 75 ms), by looking at the normalized hit rate averaged across subjects for electrode Fz (see Materials and Methods). We found that the phase accounted for 11.62% of the performance modulation (Fig. 6*C*, left hand side). As a comparison, the results from Busch et al. (2009) have also been replotted here. In that experiment, after realigning phase bins across subjects to align maximal performance at zero phase (a procedure which was unnecessary in the present experiment), the ongoing EEG phase at 7 Hz, −120 ms before stimulus onset explained 15.62% of the performance fluctuations (Fig. 6*C*, right hand side).

To summarize, in this experiment, we presented a method to study the dynamics of ongoing oscillations’ influence on perception, through the use of WN sequences. We found that the phase of θ-band ongoing oscillations is causally linked to the detection of near-perceptual threshold targets. This rhythmic influence of θ oscillations extended in time up to 200 ms after target onset, but was the largest at ∼75 ms after stimulus and could explain up to ∼12% of the performance modulations.

## Discussion

In this paper, we sought to reconcile the apparent gap between prestimulus EEG phase effects reported in the literature and the idea of phase as actively shaping perception.

In a first section, we used simulations to show that a phase modulation, artificially introduced 40 ms after stimulus onset, could be reliably detected in the prestimulus period. This depended on the ERP time-frequency content. In fact, we found that, at lower frequencies, the peak phase difference between conditions happened up to 200 ms before the true phase modulation.

The measured latencies for a phase modulation introduced at 7.08 Hz are consistent with the results of Busch et al. (2009), who found an effect of the ∼7-Hz EEG phase on the detection of near-threshold peripheral stimuli about −120 ms before stimulus onset. Of note, the 7.08-Hz simulation resulted in a median latency of −79 ms (95% CI: −88.5 to −69.5 ms) with 48/100 simulated datasets having a significant POS at −120 ms. Turning our result around, the prestimulus latency observed by Busch et al. (2009; −120 ms) could in fact be compatible with the notion that the critical phase for stimulus detection was the phase that occurred during (rather than before) stimulus processing.

It is important to highlight that the latencies reported here via simulations should not be used as a definite guide to determine the latency of the true phase modulation in a new EEG experiment. This cannot be done, because various parameters in the new EEG experiment would differ from those used in our simulations, such as the exact ERP shape on each trial, the parameters of the filters used, or the true latency at which the phase modulation occurs in the brain. All these could have a large impact on the measured latency of the phase modulation. In other words, this simulation was only intended as a proof of concept and cannot serve as an exhaustive or quantitative evaluation.

The implications of our simulations are twofold. First, any study of phase modulation of perception involving sizeable stimulus-evoked activity (that is most, if not all, studies) should be susceptible to temporal distortions of the oscillatory phase effects. Second, the numerous previous reports of prestimulus phasic influences on perception (such as Busch et al., 2009; Mathewson et al., 2009) may still be compatible with a true latency of phase modulation at or even after the stimulus onset.

Although the evoked activity leads to a temporal distortion of oscillatory activity, the true latency of phase modulations cannot be retrieved, in our case, by simply subtracting the stimulus-evoked activity (i.e., the ERP) from the EEG signal –although this approach has been successfully used in other studies (Tallon-Baudry and Bertrand, 1999) to untangle the relative contribution of “evoked” (i.e., phase locked) and “induced” oscillations (i.e., nonphase locked). In our case, the postulated phase modulation effects are actually phase-locked to the stimulus onset (i.e., we hypothesize that all hits tend to show a similar oscillatory phase relative to stimulus onset, and likewise for the misses). Subtracting condition-specific ERPs from each trial would effectively flatten any systematic phase differences between the two groups. On the other hand, simply subtracting the common ERP for hits and misses from single-trial data are also not a viable option, because it is likely to introduce artefactual phase opposition when the ERPs between conditions differ.

In a second section, we presented an experimental method based on linear-systems analysis methods (Ringach and Shapley, 2004; Lalor et al., 2006; VanRullen and Macdonald, 2012): the WN paradigm. This paradigm effectively allows us to bypass the effects of the target-evoked ERP. Thus, we could estimate, for the first time, the true latency at which ongoing EEG oscillations influence perception. Using the EEG IRF (VanRullen and Macdonald, 2012) to WN luminance sequences, we reconstructed (rather than recorded) the brain activity of subjects to new sequences, by convolving the IRF with the stimuli sequences. The reconstructed EEG allowed us to estimate the ongoing (background) oscillations before and after each presented target without the evoked response which could have biased our ability to measure the phase modulation (as shown in our simulations).

We found that the reconstructed EEG is a (relatively) good model of the recorded EEG: the two signals were significantly correlated, with the highest correlation in the α- and θ-bands. The coefficients were relatively small, meaning that at most *r* ^2^ = 0.163^2^ = 2.7% (at the maximum channel in the α-band) of the variance of the EEG signal was driven by the luminance sequence in a way that we could predict with the IRFs.

On the one hand, it might seem that this is a very small amount of variance explained. For example, using a similar approach, Kayser et al. (2015) predicted the stimulus-driven response of neurons to auditory stimulation (Kayser et al., 2015). They constructed various models of spike production based on linear spatiotemporal-response filters but also added various nonlinear factors on the prediction (Kayser et al., 2015). The best model, which included phase dependent variations in the sensory gain and the background firing of cells, could explain up to 30% of the variance (*r* ^2^) in the original signal. This increase of variance explained (relative to the present findings) can be attributed to the method used: LFP and multi-unit activity recordings are less subject to noise than scalp measurements like EEG. In fact, Lalor (2009), who also evaluated correlation strength between reconstructed and recorded EEG using the VESPA as a model for brain activity, found correlation values similar to ours: he reported a mean correlation coefficient of 0.084 over channel Oz (Lalor, 2009). Moreover, using a nonlinear (quadratic) model for the VEP only marginally improved the amount of signal explained by the model (mean *r* across subjects of 0.097; Lalor, 2009).

On the other hand, it is important to remember, that EEG is a noisy method of recording, especially when looking at single-trial data (Picton et al., 2000). The small amount of signal variability explained in the present and Lalor’s experiments (Lalor, 2009) might thus be due to the inherently noisy nature of EEG recordings. The rest of the EEG signal variance could reflect other cognitive functions (such as endogenous attention or arousal levels) or noise in the system, in the EEG recording or in the EEG reconstruction procedure. Of more interest is the fact that the IRF-based models reported here are on par with ERP-based models, explaining just as much variability in the signal, if not more. ERPs are however routinely used in experiments and considered a meaningful measure of brain activity.

Finally, despite the apparently small percentage of variance of the original EEG signal explained in our study, it could well be that this small portion of the signal is the only oscillatory activity whose phase actually modulates visual perception. In other words, we may only be able to predict a small portion of the recorded EEG signal, but our prediction was sufficiently accurate to capture most of the existing relation between EEG oscillatory phase and perception (Fig. 6*C*, ∼12% modulation in our case, compared with 16% modulation in the study by Busch et al., 2009). Using the reconstructed EEG, we found that the phase of θ (∼6 Hz) oscillations was related to the detection of near-perceptual threshold targets, as suggested before by Busch et al. (2009). But crucially, the phase between detected and missed targets was significantly different from 50 to 150 ms after stimulus presentation, with a peak at 75 ms over fronto-occipital channels. This new approach thus allowed us to uncover the true latency at which ongoing EEG oscillatory phase influences visual target detection. This could not be done before, as the presence of the ERP usually biases the detection of phase effects toward prestimulus time windows, as demonstrated in the first section. This, however, is not a problem within the present approach because only the ongoing oscillations are modeled, not the ERP.

This paradigm allowed us to explore the true latency of the effect of oscillatory phase, unbiased by evoked responses, but it also opens up a wide range of avenues of investigation. For example, it would be possible to investigate the effect of stimulus-driven ongoing oscillation amplitude on perception. In particular, this could help us untangle the contributions from stimuli driven versus top-down driven oscillations in different tasks. Indeed in our paradigm, only the ongoing oscillations directly driven by the stimuli are modeled, and thus we could evaluate their influence on performance, and compare this to the performance modulation of actually recorded EEG activity, in which top-down effects are also present.

Interestingly, this paradigm can also provide a bridge between EEG findings and other findings linking spiking activity to ongoing oscillations at the level of local neuronal populations. If the oscillations reflect the excitatory state of the population, the phase that should matter is the one expressed in the cortex at the exact moment when the stimulus information is processed. In fact, our results support this hypothesis. Further, our study lends support to the hypothesis that perception is rhythmic, and that ongoing oscillatory phase marks the underlying sampling mechanism of the environment. Here, the phase of the reconstructed EEG influences perception in a causal manner: it shapes our visual world as soon as the target enters the brain.

## Synthesis

Reviewing Editor: Bradley Postle, University of Wisconsin Decisions are customarily a result of the Reviewing Editor and the peer reviewers coming together and discussing their recommendations until a consensus is reached. When revisions are invited, a fact-based synthesis statement explaining their decision and outlining what is needed to prepare a revision will be listed below. The following reviewer(s) agreed to reveal their identity: Jason Samaha, Saskia Haegens

The reviewers and I have looked over the two reviews, and are in accord that all the points raised here are important, and would need to be satisfactorily addressed in a revision.

Reviewer #1:

This paper provides compelling evidence from simulations that prestimulus phase-perception relationships could in fact reflect phase differences early in the post-stimulus time window. Then, using an EEG reconstruction technique which removes stimulus-evoked responses from the signal, the authors show that early post-stimulus phase does indeed differentiate detected from undetected stimuli. These findings help reconcile an apparent discrepancy in prior work as to why prestimulus phase is observed to predict perception when this relationship should be maximal during the first stages of stimulus processing. This work will surely alert future researchers to the importance of considering how evoked responses influence measures of phase opposition.

The methods are sound and are clearly explicated. My only concern is that the core of the authors argument for recovering the true latency of phase effect seems to rest on the familiar distinction between the evoked (phase locked) and induced (non-phase locked) responses, but the authors do not discuss it as such. If I understand, it is the ERP that contaminates the estimation of phase opposition and when it is removed (as in the simulations, e.g., fig 2) then the true latency is revealed. It would seem that a simpler solution would be to run the phase opposition measures only on the induced component of the data, for example, by subtracting the ERP from individual trials.

Does the data from Busch et al 2009 reveal post-stimulus phase opposition once the ERP is removed? What about the data from the first session of the present experiment? Although the EEG reconstruction analysis is certainly innovative, it does not seem like a flexible enough paradigm to apply in all situations where phase opposition would be of interest. If analyzing only the induced activity produced similar results this would be an easily implemented analysis for almost any paradigm. If this approach fails, however, then some discussion of it would still be helpful for someone like me who came away from the paper impressed, but thinking that there was a more general solution to isolating the non-phase locked activity that had been missed.

A few minor points:

The data are interpreted, several times, as providing evidence for a causal influence of phase on perception, although I am not sure how the claim of causality is justified given the correlational nature of the design and analysis.

In the second session when the luminance just prior and after the stimulus is kept constant, how does this not signal the presence of the target to the subject? I guess there are a number of no target trial to estimate false alarm rates, but I couldn't find mention of them in the methods or how frequent they were.

Reviewer #2:

The authors seek to resolve the puzzling fact that most of the “pre-stimulus phase influences perception” observations in the literature are reported to peak in the order of 100 ms prior to target onset (rather than when the stimulus is actually being processed). Using simulations, as well as a clever way of reconstructing EEG signals so as to avoid the disruptive effect of ERP on phase analysis, they show that the actual critical phase for perception is a frontal/occipital theta oscillation around 75 ms after target onset, i.e., when effective processing conceivably takes place.

I think this is a really interesting study, carefully executed, and resolving an outstanding issue that has puzzled the field. The manuscript was a pleasure to read. My comments mostly pertain to issues of clarification.

1) While the reported results are pretty intriguing, reading the manuscript I was puzzled by the fairly low correlation between reconstructed and recorded EEG, with a max r of .16 for alpha, meaning about 3% explained variance (and even less for the other rhythms/sites). This signal explains ~12% modulation of behaviour. To be fair, the authors extensively discuss this and raise some important points, but I'm still trying to understand the meaning of this finding. Do the authors propose that if there was no noise in the EEG, that more behavioural variance could be explained based on these same prestimulus phase effects, or that the remaining variance is explained by other mechanisms?

Also, regarding the comparison to the Busch et al study (which reports ~16% modulation), wasn't that effect based on spontaneous prestimulus phase fluctuations, while the current one is based on oscillations directly driven by the (ongoing) stimulus sequence (i.e., the white noise stream)? Do the authors think this reflects the same underlying mechanism? I would argue that the “spontaneous” part is not exactly captured in the current model.

2) page 17 lines 11-14, the “even” vs “only” phrasing almost suggests as if this is a surprise. I would rephrase to just point out that the analysis window varied from 500 ms length (2 cycles of 250 ms for 4 hz) down to 80 ms, and that this closely matches the observed latency shift. To fully drive the point home, the authors could even consider correlating the latencies from fig2A with the window length (or alternatively, do a t-test on latency vs half window length - not sure if that would work though), rather than rely on visual appreciation of the effect.

Related to that, it might be insightful to also make this explicit for the comparison with the Busch et al. study (page 23 last paragraph and page 26). Not sure if this holds up, but if for 7 Hz 2 cycles were used (?) that would mean half window length of ~140 ms, 3 cycles would be ~215 ms. How does that match the difference in latency of the effect (-120 ms in the original study, +75 here)? I understand there wouldn't be a perfect match, given actual latencies in the brain, shape of ERP, etc, but making this explicit might be a bit more insightful for the reader.

3) Why did phase bins have to be realigned for recorded but not reconstructed EEG? Maybe I am missing the point, but the reconstructed EEG was computed per subject, so why is similar inter-subject variance not present in those data?

4) While overall the methods section is pretty clear, there is a bit of (unnecessary) mixing of results and methods, in that some results are already brought up in the methods section. e.g., page 13 lines 4-5. I would suggest to leave those reports for the results section. In the same light, figures are not discussed in order, which is a bit confusing (e.g., figure 5 is discussed before fig 2, fig 6 before fig 4, etc.), lot of jumping around there. Maybe just refrain from referring to result figures in the methods section altogether, and stick to the methods only there.

5) just for clarification: page 6 line 2, here baseline ITPC means ITPC for the combined conditions, right? not ITPC on the actual baseline window?

6) page 7 lines 16-17, was the freq of interest varied over trials? (i.e, one freq per trial?) or over simulations? This is not clear from the text. Since I did not get that part, i also don't know how to interpret the “48/100” on page 23 line 22.

7) page 12 line 23: what is meant with “shuffling the surrogate rank”?

8) figure 2 caption could be a bit more straightforward:

first describe panel A: with ERP

then B: the ERP, which was included in A (saying that it was added to A is confusing, one could read it the wrong way around).

then C: similar as A but without ERP

9) page 16 sentence starting at line 20, I suggest rewriting this sentence for clarity.

10) page 17 line 10 and 16, I would use “window function” or “analysis window” rather than “filtering window”, for clarity.

11) typo page 11 line 6, reminder > remainder

page 17 line 15, comforts > confirms?

## References

[B1] Ahumada AJ (2002) Classification image weights and internal noise level estimation. J Vis 2:121–131. 10.1167/2.1.8 12678600

[B2] Brainard DH (1997) The psychophysics toolbox. Spat Vis 10:433–436. 10.1163/156856897X00357 9176952

[B3] Busch NA, Dubois J, VanRullen R (2009) The phase of ongoing EEG oscillations predicts visual perception. J Neurosci 29:7869–7876. 10.1523/JNEUROSCI.0113-09.2009 19535598 PMC6665641

[B4] Busch NA, VanRullen R (2010) Spontaneous EEG oscillations reveal periodic sampling of visual attention. Proc Natl Acad Sci USA 107:16048–16053. 10.1073/pnas.1004801107 20805482 PMC2941320

[B5] Callaway E, Yeager CL (1960) Relationship between reaction time and electroencephalographic alpha phase. Science 132:1765–1766. 13689987 10.1126/science.132.3441.1765

[B6] Chakravarthi R, VanRullen R (2012) Conscious updating is a rhythmic process. Proc Natl Acad Sci USA 109:10599–10604. 10.1073/pnas.1121622109 22689974 PMC3387058

[B7] Delorme A, Makeig S (2004) EEGLAB: an open source toolbox for analysis of single-trial EEG dynamics including independent component analysis. J Neurosci Methods 134:9–21. 10.1016/j.jneumeth.2003.10.009 15102499

[B8] Drewes J, VanRullen R (2011) This is the rhythm of your eyes: the phase of ongoing electroencephalogram oscillations modulates saccadic reaction time. J Neurosci 31:4698–4708. 10.1523/JNEUROSCI.4795-10.2011 21430168 PMC6622921

[B9] Dugué L, Marque P, VanRullen R (2011) The phase of ongoing oscillations mediates the causal relation between brain excitation and visual perception. J Neurosci 31:11889–11893. 10.1523/JNEUROSCI.1161-11.2011 21849549 PMC6623205

[B10] Fries P (2005) A mechanism for cognitive dynamics: neuronal communication through neuronal coherence. Trends Cogn Sci 9:474–480. 10.1016/j.tics.2005.08.011 16150631

[B11] Fries P, Schröder J-H, Roelfsema PR, Singer W, Engel AK (2002) Oscillatory neuronal synchronization in primary visual cortex as a correlate of stimulus selection. J Neurosci 22:3739–3754. 11978850 10.1523/JNEUROSCI.22-09-03739.2002 PMC6758402

[B12] Haegens S, Barczak A, Musacchia G, Lipton ML, Mehta AD, Lakatos P, Schroeder CE (2015) Laminar profile and physiology of the α rhythm in primary visual, auditory, and somatosensory regions of neocortex. J Neurosci 35:14341–14352. 10.1523/JNEUROSCI.0600-15.2015 26490871 PMC4683691

[B13] Haegens S, Nácher V, Luna R, Romo R, Jensen O (2011) α-Oscillations in the monkey sensorimotor network influence discrimination performance by rhythmical inhibition of neuronal spiking. Proc Natl Acad Sci USA 108:19377–19382. 10.1073/pnas.1117190108 22084106 PMC3228466

[B14] Hanslmayr S, Volberg G, Wimber M, Dalal SS, Greenlee MW (2013) Prestimulus oscillatory phase at 7 Hz gates cortical information flow and visual perception. Curr Biol 23:2273–2278. 10.1016/j.cub.2013.09.020 24184106

[B15] Jacobs J, Kahana MJ, Ekstrom AD, Fried I (2007) Brain oscillations control timing of single-neuron activity in humans. J Neurosci 27:3839–3844. 10.1523/JNEUROSCI.4636-06.2007 17409248 PMC6672400

[B16] Kayser C, Wilson C, Safaai H, Sakata S, Panzeri S (2015) Rhythmic auditory cortex activity at multiple timescales shapes stimulus-response gain and background firing. J Neurosci 35:7750–7762. 10.1523/JNEUROSCI.0268-15.2015 25995464 PMC4438125

[B17] Lachaux J, Rodriguez E, Martinerie J, Varela FJ (1999) Measuring phase synchrony in brain signals. Hum Brain Mapp 8:194–208. 10619414 10.1002/(SICI)1097-0193(1999)8:4<194::AID-HBM4>3.0.CO;2-C PMC6873296

[B18] Lakatos P, Karmos G, Mehta AD, Ulbert I, Schroeder CE (2008) Entrainment of neuronal oscillations as a mechanism of attentional selection. Science 320:110–113. 18388295 10.1126/science.1154735

[B19] Lakatos P, Shah AS, Knuth KH, Ulbert I, Karmos G, Schroeder CE (2005) An oscillatory hierarchy controlling neuronal excitability and stimulus processing in the auditory cortex. J Neurophysiol 94:1904–1911. 15901760 10.1152/jn.00263.2005

[B20] Lalor EC (2009). Modeling the human visual system using the white-noise approach. In: Proceeding of the 4th International IEEE/EMBS Conference on Neural Engineering, Antalya, Turkey, pp 589–592.

[B21] Lalor EC, Pearlmutter BA, Reilly RB, McDarby G, Foxe JJ (2006) The VESPA: a method for the rapid estimation of a visual evoked potential. NeuroImage 32:1549–1561. 10.1016/j.neuroimage.2006.05.054 16875844

[B22] Marmarelis P, Marmarelis VZ (1978). The white noise method of system identification In: Analysis of physiological systems: the white-noise approach. New York: Plenum Press.

[B23] Mathewson KE, Gratton G, Fabiani M, Beck DM, Ro T (2009) To see or not to see: prestimulus alpha phase predicts visual awareness. J Neurosci 29:2725–2732. 10.1523/JNEUROSCI.3963-08.2009 19261866 PMC2724892

[B24] McGill R, Tukey JW, Larsen WA (1978) Variations of box plots. Am Stat 32:12–16. 10.2307/2683468

[B25] McLelland D, Lavergne L, VanRullen R (2016) The phase of ongoing EEG oscillations predicts the amplitude of peri-saccadic mislocalization. Sci Rep 6:29335 . 10.1038/srep29335 27403937 PMC4941415

[B26] Nunn CM, Osselton JW (1974) The influence of the EEG alpha rhythm on the perception of visual stimuli. Psychophysiology 11:294–303. 4421317 10.1111/j.1469-8986.1974.tb00547.x

[B27] Picton TW, Bentin S, Berg P, Donchin E, Hillyard SA, Johnson R Jr, Miller GA, Ritter W, Ruchkin DS, Rugg MD, Taylor MJ (2000) Guidelines for using human event-related potentials to study cognition: recording standards and publication criteria. Psychophysiology 37:127–152. 10.1111/1469-8986.3720127 10731765

[B28] Ringach D, Shapley R (2004) Reverse correlation in neurophysiology. Cogn Sci 28:147–166. 10.1207/s15516709cog2802_2

[B29] Tallon-Baudry C, Bertrand O (1999) Oscillatory gamma activity in humans and its role in object representation. Trends Cogn Sci 3:151–162. 10322469 10.1016/s1364-6613(99)01299-1

[B30] Tallon-Baudry C, Bertrand O, Delpuech C, Pernier J (1996) Stimulus specificity of phase-locked and non-phase-locked 40 Hz visual responses in human. J Neurosci 16:4240–4249. 8753885 10.1523/JNEUROSCI.16-13-04240.1996 PMC6579008

[B31] VanRullen R (2011) Four common conceptual fallacies in mapping the time course of recognition. Front Psychol 2:365. 10.3389/fpsyg.2011.00365 22162973 PMC3232460

[B32] VanRullen R (2016a) How to evaluate phase differences between trial groups in ongoing electrophysiological signals. Front Neurosci 10:426 10.3389/fnins.2016.00426 27683543 PMC5021700

[B33] VanRullen R (2016b) Perceptual cycles. Trends Cogn Sci 20:723–735. 10.1016/j.tics.2016.07.006 27567317

[B34] VanRullen R, Koch C (2003) Is perception discrete or continuous? Trends Cogn Sci 7:207–213. 12757822 10.1016/s1364-6613(03)00095-0

[B35] VanRullen R, Macdonald JSP (2012) Perceptual echoes at 10 Hz in the human brain. Curr Biol 22:995–999. 10.1016/j.cub.2012.03.050 22560609

[B36] Watson AB, Pelli DG (1983) QUEST: a Bayesian adaptive psychometric method. Percept Psychophys 33:113–120. 10.3758/BF03202828 6844102

